# Epigenetic memory in the context of nuclear reprogramming and cancer

**DOI:** 10.1093/bfgp/elt011

**Published:** 2013-04-12

**Authors:** Richard P. Halley-Stott, John B. Gurdon

**Keywords:** epigenetic memory, nuclear reprogramming, cancer, histone modifications, DNA methylation

## Abstract

Epigenetic memory represents a natural mechanism whereby the identity of a cell is maintained through successive cell cycles, allowing the specification and maintenance of differentiation during development and in adult cells. Cancer is a loss or reversal of the stable differentiated state of adult cells and may be mediated in part by epigenetic changes. The identity of somatic cells can also be reversed experimentally by nuclear reprogramming. Nuclear reprogramming experiments reveal the mechanisms required to activate embryonic gene expression in adult cells and thus provide insight into the reversal of epigenetic memory. In this article, we will introduce epigenetic memory and the mechanisms by which it may operate. We limit our discussion primarily to the context of nuclear reprogramming and briefly discuss the relevance of memory and reprogramming to cancer biology.

## INTRODUCTION

Differentiation is remarkably stable. Differentiated cells almost never switch identity. This stability in phenotypic identity, both during development and in adult cells, is achieved by ensuring stable gene expression profiles whereby only the correct genes for any particular cell type will be expressed. The nature of this stability is controlled, at least in part, by epigenetic mechanisms in the form of DNA methylation, histone variants and the posttranslational modification of histones.

Maintenance of these epigenetic systems is achieved across multiple cellular generations by a form of ‘memory’ where prior epigenetic state and thus gene expression state is maintained throughout development, generating the stable adult tissues of the adult organism. This review will initially introduce epigenetic memory (focussed predominantly on epigenetic mechanisms that involve the covalent modification of DNA and histone proteins) and discuss this phenomenon in the context of nuclear reprogramming and its potential relevance to cancer.

### What do we mean by epigenetic memory?

Epigenetics is the study of heritable changes in gene expression or cellular phenotype caused by mechanisms other than changes in the underlying DNA sequence [1]. This refers to functionally relevant modifications to gene expression that do not involve a change in nucleotide sequence.

The term ‘epigenetic’ has been taken to mean different things at different times since its original inception [2] and definitions often extend to include the mechanisms by which epigenetic phenomena are manifested. Examples of these mechanisms include the action of transcription factors (particularly those considered to be pioneer and bookmarking factors), noncoding RNAs, covalent modification of DNA and histone proteins (with chemical motifs such as methylation groups), and the action of the agents that create these modifications, as well as external signalling molecules.

Loss of the stable gene expression that maintains cellular phenotype is extremely serious for the organism and can, amongst other things, lead to the formation of cancers. Understanding how epigenetic mechanisms establish, maintain and control gene expression is important both for the field of developmental biology and cancer biology.

Adult cells may also be induced to express embryonic genes and silence their somatic genes under certain experimental conditions—a process known as nuclear reprogramming. This phenomenon was first demonstrated by nuclear transplantation in *Amphibia* [3] and has been reproduced in a range of taxa including many mammalian species [4, 5]. Reversal of differentiation and transversion of cell identity have also been achieved by fusing cells to form heterokaryons [6]. Reprogramming has also been shown possible, without physical manipulation, by the overexpression of four embryonic master regulatory transcription factors in somatic cells, leading to the production of induced pluripotent stem (iPS) cells [7].

Reprogramming somatic cells provides investigators with a tool to understand what changes need to be made to an adult cell to erase stable somatic gene expression and activate the embryonic gene expression programs. Further to this, when reprogramming of transcription is incomplete, it may be indicative of epigenetic memory.

### What do we mean by epigenetic memory in the context of nuclear reprogramming experiments?

We define ‘epigenetic memory’ as the retention of gene transcription patterns in a nucleus after the induction of new gene expression has occurred. This induction could result from differentiation or reprogramming signals such as those that follow the transplantation of a nucleus into an egg. If epigenetic memory persists, there will be transcription of genes that are reminiscent of that of the original cell type; this transcription pattern will persist despite the induction of new transcription (and gene silencing) in the introduced nucleus by the egg. Critically, the persistence of silenced or active genes occurs without genetic change and must be the result of an epigenetic mechanism.

## SHORT-TERM MEMORY: ONE CELL TO ITS DAUGHTER CELLS

An essential facet of epigenetics or ‘epigenetic memory’ is that a gene expression state must be maintained or re-established through each cell cycle. As such, an ‘epigenetic state’ must be maintained through both S-phase and mitosis. These are two very different cellular processes, both of which could lead to epigenetic erasure.

During S-phase, genomic DNA must be unpacked, replicated and repacked, in a manner that faithfully replicates not only the sequence itself but also the other information systems relating to gene expression present at any particular genomic location. By this, we mean DNA methylation patterns, histone modifications and nucleosome positions, and we include other chromatin-binding proteins such as the heterochromatin and polycomb proteins. If these are not faithfully replicated on both daughter strands, there is a potential for a change in the transcriptional activity of that location, something that may lead to undesirable consequences for the cell.

Likewise, the ‘epigenetic profile’ of a given locus must be faithfully re-established following mitosis. In addition to alteration of nucleosome positioning as a cell transits through mitotic division [8], there are a number of posttranslational histone modifications that are either maintained or changed, and which may impact gene expression after mitotic exit [9–12]. Additionally, the many non- ‘core chromatin’ proteins that are ejected from mitotic chromosomes will need to find their way back to the correct location at mitotic exit. Indeed, mitosis has been identified as a critical stage when a shift in gene expression between cellular generations may be achieved by changing the complement of chromatin-binding proteins present in a cell [13, 14].

This persistence of an ‘epigenetic state’, be it true persistence or continual re-establishment after DNA replication or mitosis, is undoubtedly a form of natural memory; it is presumably designed to prevent unintended changes in gene expression at these times, thus maintaining the stability of phenotypic state seen in differentiated tissue.

## MEDIUM TERM: MEMORY THROUGH WHOLE LINEAGES FROM AN EMBRYONIC CELL TO DIFFERENTIATED TISSUE

The longer term consequence of maintaining an epigenetic state through each cell cycle is that the persistence of epigenetic states throughout development allows the gradual specification of adult tissue types. By restricting developing cells to particular lineages through the restriction of gene expression in a lineage-specific manner, embryonic tissues become increasingly specialized, ultimately giving rise to the highly differentiated and ordered cell types of the adult body plan. It is this ‘epigenetic restriction’ of gene expression that provides a mechanistic basis to Waddington’s ‘Canalization of development’ [15], often referred to as ‘Waddington’s epigenetic landscape’ [16].

Following the establishment of differentiation, cells almost never change their ‘epigenetic state’ or revert to a gene expression state typical of an earlier stage of less differentiation. An exception to this statement is the onset of cancer.

## LONG TERM: FROM ONE GENERATION TO THE NEXT—THROUGH MEIOSIS

On a much longer time scale, there are examples of epigenetic states that transit through multiple generations of individuals. In this case, the memory persists through meiosis, allowing transmission of the state from one adult generation to the next. A well-known example of this is paramutation of the R locus in maize, where one allele is able to induce epigenetic changes in another, leading to heritable changes in seed coloration [17]. The mechanism behind this gene expression change is the induction of DNA methylation in the paramutable allele [18, 19] which is then maintained through meiosis and transmitted to subsequent generations. A recent example of this trans-generational inheritance showed inheritance of gene silencing in worms across 24 generations. This has been demonstrated to be epigenetically controlled and is maintained in the absence of the piRNA inducing signal [20].

## POSSIBLE MECHANISMS OF EPIGENETIC MEMORY

**Figure 1:**
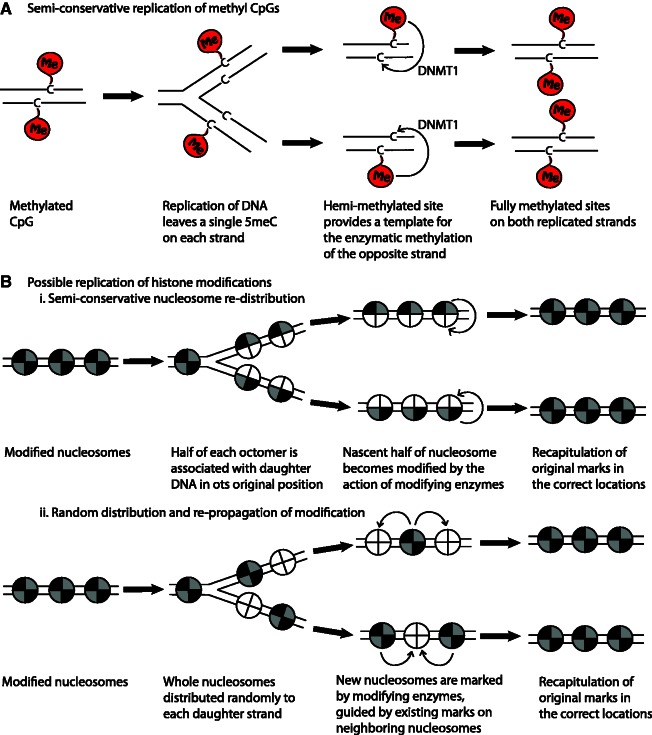
Mechanisms that may maintain epigenetic memory through DNA replication. (**A**) DNA methylation (‘me’ bubbles on cytosine bases, represented by ‘C’, on DNA strands which are represented by a black lines) is maintained by a mechanism of semi-conservative replication. After replication of the DNA strands, hemi-methylated CpG sites become fully methylated by the action of DNMT1. The DNA methylation pattern in the paternal strand provides a template for methylation of the nascent strand. (**Bi**) Epigenetic information on nucleosomes (represented here as quartered balls, with each of the four histones as a quarter of the ball, on a DNA strand, represented by black lines) may be transmitted to each of the daughter DNA strands during DNA replication by semi-conservative distribution of half of the nucleosome to each new DNA molecule. Unmodified histones (white quarters) are then incorporated with the old histones to make up a full nucleosome with half of the octomer marked. The histone marks are then copied to the new histones within each nucleosome by the action of histone-modifying enzymes. (**Bii**) An alternative hypothesis to semi-conservative nucleosome replication suggests that marked nucleosomes are randomly associated with each of the new DNA strands, becoming interspersed at random with new nucleosomes without any markings. Epigenetic information will then be transmitted to new nucleosomes by the action of histone modifying enzymes using marked neighboring nucleosomes as a template.

For epigenetic memory to be propagated on any of these three time scales, the epigenetic decoration of gene loci must be maintained. At the simplest level, DNA methylation at genomic loci is maintained through cell generations and in the case of certain imprints through meiosis by semi-conservative replication, where hemi-methylated sites lead to methylation of the opposite strand by the action of DNA methyl transferase 1 (DNMT1) and possibly other enzymes (Figure 1A). The mechanisms of DNA methylation maintenance have been extensively reviewed elsewhere [21].

Less is understood about how epigenetic information carried on nucleosomes, in the form of post-translational histone modifications and variants, is transmitted to each of the chromatin fibres following DNA replication.

Currently, three models exist, which are briefly described later as they have been extensively reviewed recently by others [22–26]. The simplest explanation for the propagation of epigenetic information on histones is a semi-conservative approach where half of each old nucleosome is partitioned to each of the daughter DNA strands, where it is joined by nascent histones to complete a full nucleosome (Figure 1Bi). The information carried on the old histone is thereby copied to the nascent histones within the same nucleosome by the action of histone modifying enzymes. The second model suggests that old nucleosomes are randomly distributed to each of the daughter DNA strands. Nascent nucleosomes are incorporated into the daughter strands at the same time, such that on both daughter strands there is a random assortment of old histones, carrying epigenetic information, and new ones without this information. Post-translational modifications are then transmitted to the histones of neighbouring nucleosomes to recapitulate the original modification patterns (Figure 1Bii). The final model concerns itself with the persistence of the inducing signal and not the modification itself, suggesting that the ‘epigenetic modifications’ are re-established within each cell cycle. There is some recent evidence for this model, at least in certain instances, where it has been demonstrated that polycomb and trithorax group histone-modifying enzymes remain closely associated with their target genomic loci following DNA replication in *Drosophila* embryo cells. These enzymes then add histone marks to these nascent nucleosomes in the same positions as was seen prior to DNA replication [27]. This final model suggests that as long as the enzymes and targeting mechanisms (activators, repressors and non-coding RNAs for example) that establish an ‘epigenetic state’ are segregated to both daughter cells, then histone decoration can be re-established in a site-specific manner, *de novo,* with each cell cycle and thus ‘memory’ involves only the continued production of inducing signals.

There is however much evidence also suggesting that once an ‘epigentic state’ is established, the state will persist without the continued influence of the original inducing signal. There are many natural examples of this in normal development such as the irreversible silencing of the X-chromosome in the absence of the silencing signal, the long non-coding RNA Xist [28]. An early experimental demonstration of this was shown by the conversion of cultured 10T1/2 cells into muscle cells by 5-aza-2′-deoxycytidine treatment [29], an agent that leads to demethylation of methylated CpG sites. Critically, the phenotypic changes were maintained after the inducing signal was removed from the culture media. More recent examples include the persistence of ectopically (sequence-independent) induced centrosomes through many cell divisions, long after the removal of the inducing signal in *Drosophila* cells [30].

Maintenance or re-establishment of correct gene expression after mitotic exit presents another challenge to the cell. As noted earlier, mitotic exit is a time period where the gene expression pattern of daughter cells may be changed from the parent cell by supplying new DNA-binding proteins to associate with genomic loci following chromatin decondensation. One mechanism to prevent this from occurring is known as ‘mitotic bookmarking’. Certain transcription factors will remain associated with specific loci throughout mitosis when most other factors are displaced from chromosomes (reviewed in [31, 32]). An example of this is the retention of the hematopoietic transcription factor GATA1 and chromatin proteins such as MLL and BRD4 at specific loci during mitosis. These bound loci are rapidly activated on mitotic exit, maintaining continued expression of ‘housekeeping’ and lineage-specific genes in daughter cells [33–35].

Likewise, gene-regulatory information may be ‘bookmarked’ by maintaining certain histone modifications (and the readers and writers of these in some instances) at specific loci (reviewed in [36]). Finally, DNA methylation would also serve as a ‘bookmark’, with the information carried by this modification passing to each daughter cell.

## EXAMPLES OF EPIGENETIC MEMORY IN NUCLEAR REPROGRAMMING

**Figure 2:**
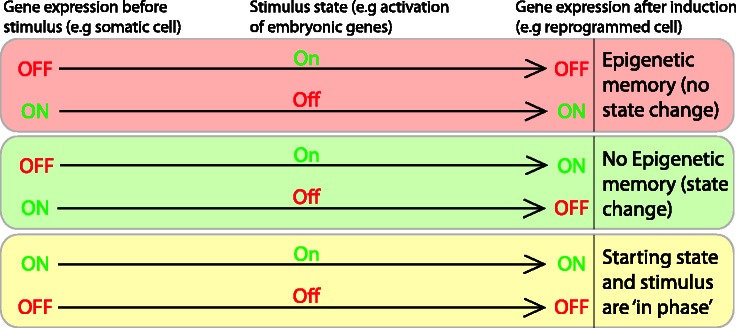
Epigenetic memory is revealed by reprogramming experiments. Memory is revealed by nuclear reprogramming experiments when the transcriptional state of a particular gene fails to change to the induced state (top box). An example of this is the failure to activate pluripotency genes, which are ‘off’ in somatic cells, following an inducing signal to activate these genes (such as the Yamanaka reprogramming factors). In contrast, genes are competent for reprogramming if they correctly change to the induced state, in which case no memory is seen (middle box). If the transcription state of a gene is the same as the induction state (‘in phase’) then no change will be observed (bottom box).

The controlled induction of embryonic gene expression in differentiated cells by nuclear reprogramming drives a shift in somatic transcription patterns to that of an embryonic pattern. When these changes in gene expression fail to occur, or occur incompletely, it is indicative of mechanisms involved in maintaining epigenetic memory (Figure 2). As a consequence, nuclear reprogramming provides a unique opportunity to study epigenetic memory and more importantly the mechanisms that maintain stable repression or expression of genes. This kind of memory has now been demonstrated in each of the major reprogramming methodologies.

Misregulated gene expression was described some time ago in embryos from mammalian nuclear transfers when compared with naturally fertilized embryos [37–41]. This phenomenon may well be the result of epigenetic memory and this was formally demonstrated in this context using amphibian nuclear transfers, where ectopic expression of lineage markers is seen in cloned embryos. Moreover, the identity of these markers is related to the tissue origin of the donor nucleus, suggesting a failure during reprogramming to silence somatic lineage genes [42]. This is explained, at least in part, by an unusually high concentration of histone H3.3 in recipient eggs, which appears to maintain ‘on’ genes in an on state [43]. Failure to fully erase or correctly introduce other epigenetic factors in cloned embryos has also been extensively demonstrated—most notably in the case of DNA and histone methylation and acetylation [44–49]. In line with this, there have been several reports showing improved success when agents that modify epigenetic signatures are employed during nuclear reprogramming by nuclear transfer. The use of histone deacetylase inhibitors (such as Trichostatin A) have both shown enhanced clone generation [50, 51]. The action of these agents is presumably to assist the erasure of ‘memory marks’ following nuclear transfer.

The use of reprogramming systems to study the epigenetic memory also provides valuable insight into the mechanism by which memory is established and maintained. Recent work in cell fusion (described later), iPS and in nuclear transfer has allowed the identification of molecules involved in the maintenance of epigenetic memory. For example, nuclear transfer into GV-stage amphibian oocytes revealed that a histone H2A variant (macro-H2A) and the absence of histone acetylation are involved in maintaining the silent state of the X chromosome in donor nuclei after nuclear transfer [52, 53].

As with nuclear transfer, epigenetic memory has been clearly demonstrated when generating cells by iPS and persists long after the induction of reprogramming [54–57]. The efficiency of iPS generation appears to be improved with the use of agents that alter epigenetic signatures, such as histone deacetylase and DNA methyltransferase inhibitors [58]. As with nuclear transfer, these presumably assist reprogramming by the erasure of epigenetic memory marks which otherwise fail to be fully reprogrammed.

The presence of ‘epigenetic memory’ is also clearly demonstrated in heterokaryon-based reprogramming, whereby a number of genes that are resistant to *trans*-acting reprogramming stimuli have been identified [59]. Genome wide analysis of chromatin and DNA methylation profiles associated with genes that display this ‘occlusion’ (*cis*-mediated silencing or epigenetic memory) revealed a number of chromatin signatures that are clearly linked with genes that fail to activate following induction by cell fusion [60]. The authors here established a causal link between DNA methylation and memory but were not able to establish such a link between the majority of the other transcriptionally repressive histone marks and memory. These were however highly correlated with the observed memory effects. This surprising result may suggest that histone modifications are not definitive in establishing states of epigenetic memory. Alternatively, the result may reflect the complexity of histone modifications in controlling gene expression. The use of reprogramming systems to analyse these relationships may well lead to an enhanced understanding of what is cause and effect in terms of epigenetic control structures as has been demonstrated by Lee *et al.* [60].

## RELEVANCE TO CANCER

In a natural context, reversal of differentiation almost never occurs and when it does, can lead to disease and particularly the generation of cancers. There appear to be several similarities between cancer cells and early stem cells, such as the ability to self-renew, relaxed cell cycle checkpoint control [61, 62], hypoxic growth conditions [63], and of greatest relevance to this review, epigenetic profiles and gene expression patterns. The ectopic expression of genes involved in pluripotency, such as Oct4, in some cancers has been known for some time [64]. A number of recent publications making use of modern expression analysis techniques have greatly expanded our understanding of the kinds of embryonic genes that are commonly expressed between embryonic stem (ES), iPS and cancerous cells [65–69] and have been reviewed elsewhere recently [70].

Many cancers appear to maintain a degree of their original identity—i.e. glioma gene expression profiles continue to resemble glial cell-type expression patterns, at least in part [71]. This partial maintenance of identity can probably be attributed to an ‘epigenetic memory’ even when there has been some dedifferentiation of the somatic identity. This is reminiscent of the ‘epigenetic memory’ seen in the experimental reprogramming of somatic cells. Despite the fact that some cancerous cells may not appear to express ‘stem’ genes, there does appear to be a link between the degree of dedifferentiation, linked to aggressive cancer progression and embryonic gene expression patterns [72] .

Given that there are a number of similarities in gene expression between pluripotent cells and some cancer lines, it is relevant to ask what genes associated with pluripotency are turned on or off during carcinogenesis.

One factor that appears to be a central link between cancer cells and pluripotent cells is the expression of the proto-oncogene myc [66]. Myc is a well-known player in carcinogenesis and appears to act primarily by gene activation, inducing the expression of genes involved in cell cycle progression and metabolism, while silencing the transcription of cell cycle checkpoint genes and differentiation genes. Myc activity in carcinogenesis has been extensively reviewed (Meyer and Penn [73], and Sing and Dalton [74] for example). During early development, Myc expression is necessary for the maintenance of pluripotency through interaction with the cell cycle machinery and repressing differentiation programs [75, 76].

It is interesting that despite the observed expression of other pluripotency regulators in some cancers [72], it appears that the link between oncogenesis and pluripotency lies largely with myc [65, 66].

This link between cancer and pluripotency has been experimentally demonstrated by analysing the gene expression patterns of iPS and ‘oncogenic foci’, transformed from the same starting cells [77]. It was observed that a number of the same gene sets were either silenced or activated in both iPS and transformed cells, often targets of myc and not necessarily the core pluripotency genes.

Given the important role myc plays in oncogenesis, it would appear that a greater understanding of how myc expression is regulated, particularly in an epigenetic context, in somatic and adult stem cells is necessary.

## DISCUSSION

Epigenetic memory is a natural phenomenon involved in the maintenance of the stability of cell identity. It is through this mechanism that memory of identity is maintained during genome replication and cell division. Ultimately, allowing the progressive differentiation and specialization of embryonic cells during development to form the many somatic cell types of the adult in an ordered manner. It is also through this mechanism that cells maintain their identity and do not spontaneously switch identity or proliferate in an uncontrolled manner. As such, understanding the mechanisms that underpin epigenetic memory in normal development and in carcinogenesis will greatly assist both our understanding of development and in the identification of cancer treatment targets. The reprogramming of somatic cells to a state of embryonic gene expression provides investigators with tools to study these mechanisms and already headway is being made in identifying molecules involved in specifying epigenetic memory.

An interesting question relates to the necessity of the cell cycle to erase the epigenetic marks involved in the maintenance of memory. It would appear that both mitosis [13, 14] and S-phase appear to be times at which epigenetic patterns and gene expression programs may be extensively altered. Transition through mitosis has been suggested as a possible mechanism by which reprogramming may occur, with transcription factors that drive embryonic gene expression binding to chromatin at mitotic exit in place of somatic factors [78]. Reprogramming work with Prophase I arrested oocytes in amphibians (known as GV-stage oocytes in mammals) suggests that mitosis is not required, as no DNA replication or cell cycle progression is observed in to the donor nuclei following transplantation [79, 80]. An analogous situation may also be the case in reprogramming assays based on heterokaryon formation. Activation of pluripotent genes is seen from fibroblasts fused with ES cells in the absence of DNA replication and cell division [81, 82]. There is however, also evidence to suggest that DNA replication may augment reprogramming or even be necessary for reprogramming in heterokaryons [83].

iPS-based reprogramming suggests that, while some reprogramming events do occur without cell division, the activation of embryonic genes does require at least a few cell divisions [84]. This may potentially be to permit passive DNA demethylation [85] and cell cycling will certainly be necessary if new cell types need to be generated and expanded from just a few reprogrammed somatic cells.

Although the precise role of DNA replication and cell division in reprogramming remains unclear, it may well be necessary for the efficient erasure of memory marks. Analogously, it is of interest to know if the erasure of epigenetic memory in cancers is achieved in a cell cycle dependent manner (leading to the expression of embryonic genes) or if the increased proliferation of cancerous cells is in response to this loss of epigenetic memory (through the expression of cell cycle regulatory and embryonic genes).

**Key Points**

- Epigenetics is the study of heritable changes in gene expression caused by mechanisms other than changes in the underlying DNA sequence.
- Epigenetic memory is a natural mechanism involved in maintaining cell identity through multiple cell divisions and is thus essential in maintaining stability of cellular identity in an organism.
- Reversal of the differentiated state by nuclear reprogramming permits the identification of the mechanisms that underpin epigenetic memory.
- Cancer is a loss or reversal of a stable differentiated state and is often associated with the expression of embryonic genes, most notably myc.
- Understanding how embryonic genes are turned on in somatic cells, particularly in an epigenetic context, may provide targets for effective cancer treatments.

## FUNDING

R.P.H.-S. and J.B.G are supported by the Medical Research Council [G1001690] and the Wellcome Trust.

## References

[1] Russo VEA, Martienssen RA, Riggs AD (1996). Epigenetic Mechanisms of Gene Regulation.

[2] Haig D (2004). The (dual) origin of epigenetics. Cold Spring Harb Symp Quant Biol.

[3] Gurdon JB (1960). The developmental capacity of nuclei taken from differentiating endoderm cells of *Xenopus laevis*. J Embryol Exp Morphol.

[4] Wilmut I, Schnieke AE, McWhir J (1997). Viable offspring derived from fetal and adult mammalian cells. Nature.

[5] Cibelli J (2007). Developmental biology. A decade of cloning mystique. Science.

[6] Blau HM, Pavlath GK, Hardeman EC (1985). Plasticity of the differentiated state. Science.

[7] Takahashi K, Yamanaka S (2006). Induction of pluripotent stem cells from mouse embryonic and adult fibroblast cultures by defined factors. Cell.

[8] Komura JI (2005). Disappearance of nucleosome positioning in mitotic chromatin in vivo. J Biol Chem.

[9] Kouskouti A, Talianidis I (2005). Histone modifications defining active genes persist after transcriptional and mitotic inactivation. EMBO J.

[10] Valls E, Sánchez-Molina S, Martínez-Balbás MA (2005). Role of histone modifications in marking and activating genes through mitosis. J Biol Chem.

[11] Terrenoire E, McRonald F, Halsall JA (2010). Immunostaining of modified histones defines high-level features of the human metaphase epigenome. Genome Biol.

[12] Xu D, Bai J, Duan Q (2009). Covalent modifications of histones during mitosis and meiosis. Cell Cycle.

[13] Martínez-Balbás MA, Dey A, Rabindran SK (1995). Displacement of sequence-specific transcription factors from mitotic chromatin. Cell.

[14] Holtzer H, Rubinstein N, Fellini S (1975). Lineages, quantal cell cycles, and the generation of cell diversity. Q Rev Biophys.

[15] Waddington CH (1959). Canalization of development and genetic assimilation of acquired characters. Nature.

[16] Hochedlinger K, Plath K (2009). Epigenetic reprogramming and induced pluripotency. Development.

[17] Brink RA (1956). A genetic change associated with the R locus in maize which is directed and potentially reversible. Genetics.

[18] Walker EL (1998). Paramutation of the r1 locus of maize is associated with increased cytosine methylation. Genetics.

[19] Ronchi A, Petroni K, Tonelli C (1995). The reduced expression of endogenous duplications (REED) in the maize R gene family is mediated by DNA methylation. EMBO J.

[20] Ashe A, Sapetschnig A, Weick E-M (2012). piRNAs can trigger a multigenerational epigenetic memory in the germline of *C. elegans*. Cell.

[21] Bird A (2002). DNA methylation patterns and epigenetic memory. Genes Dev.

[22] Margueron R, Reinberg D (2010). Chromatin structure and the inheritance of epigenetic information. Nat Rev Genet.

[23] Sarkies P, Sale JE (2011). Propagation of histone marks and epigenetic memory during normal and interrupted DNA replication. Cell Mol Life Sci.

[24] Rocha W, Verreault A (2008). Clothing up DNA for all seasons: histone chaperones and nucleosome assembly pathways. FEBS Lett.

[25] Annunziato AT (2004). Split decision: what happens to nucleosomes during DNA replication?. J Biol Chem.

[26] Probst AV, Dunleavy E, Almouzni G (2009). Epigenetic inheritance during the cell cycle. Nat Rev Mol Cell Biol.

[27] Petruk S, Sedkov Y, Johnston DM (2012). TrxG and PcG proteins but not methylated histones remain associated with DNA through replication. Cell.

[28] Wutz A, Jaenisch R (2000). A shift from reversible to irreversible X inactivation is triggered during ES cell differentiation. Mol Cell.

[29] Constantinides PG, Taylor SM, Jones PA (1978). Phenotypic conversion of cultured mouse embryo cells by aza pyrimidine nucleosides. Dev Biol.

[30] Mendiburo MJ, Padeken J, Fülöp S (2011). *Drosophila* CENH3 is sufficient for centromere formation. Science.

[31] Delcuve GP, He S, Davie JR (2008). Mitotic partitioning of transcription factors. J Cell Biochem.

[32] Zaidi SK, Young DW, Montecino MA (2010). Mitotic bookmarking of genes: a novel dimension to epigenetic control. Nat Rev Genet.

[33] Kadauke S, Udugama MI, Pawlicki JM (2012). Tissue-specific mitotic bookmarking by hematopoietic transcription factor GATA1. Cell.

[34] Blobel GA, Kadauke S, Wang E (2009). A reconfigured pattern of MLL occupancy within mitotic chromatin promotes rapid transcriptional reactivation following mitotic exit. Mol Cell.

[35] Zhao R, Nakamura T, Fu Y (2011). Gene bookmarking accelerates the kinetics of post-mitotic transcriptional re-activation. Nat Cell Biol.

[36] Wang F, Higgins JMG (2012). Histone modifications and mitosis: countermarks, landmarks, and bookmarks. Trends Cell Biol.

[37] Humpherys D, Eggan K, Akutsu H (2002). Abnormal gene expression in cloned mice derived from embryonic stem cell and cumulus cell nuclei. Proc Natl Acad Sci U S A.

[38] Humpherys D (2001). Epigenetic instability in ES cells and cloned mice. Science.

[39] Kohda T (2005). Variation in gene expression and aberrantly regulated chromosome regions in cloned mice. Biol Reprod.

[40] Boiani M, Eckardt S, Schöler HR (2002). Oct4 distribution and level in mouse clones: consequences for pluripotency. Genes Dev.

[41] Bortvin A (2003). Incomplete reactivation of Oct4-related genes in mouse embryos cloned from somatic nuclei. Development.

[42] Ng RK, Gurdon JB (2005). Epigenetic memory of active gene transcription is inherited through somatic cell nuclear transfer. Proc Natl Acad Sci U S A.

[43] Ng RK, Gurdon JB (2007). Epigenetic memory of an active gene state depends on histone H3.3 incorporation into chromatin in the absence of transcription. Nat Cell Biol.

[44] Dean W, Santos F, Stojkovic M (2001). Conservation of methylation reprogramming in mammalian development: aberrant reprogramming in cloned embryos. Proc Natl Acad Sci U S A.

[45] Bourc'his D, Le Bourhis D, Patin D (2001). Delayed and incomplete reprogramming of chromosome methylation patterns in bovine cloned embryos. Curr Biol.

[46] Kang YK, Koo DB, Park JS (2001). Aberrant methylation of donor genome in cloned bovine embryos. Nat Genet.

[47] Blelloch R, Wang Z, Meissner A (2006). Reprogramming efficiency following somatic cell nuclear transfer is influenced by the differentiation and methylation state of the donor nucleus. Stem Cells.

[48] Zhang M, Wang F, Kou Z (2009). Defective chromatin structure in somatic cell cloned mouse embryos. J Biol Chem.

[49] Wee G, Koo D-B, Song B-S (2006). Inheritable histone H4 acetylation of somatic chromatins in cloned embryos. J Biol Chem.

[50] Kishigami S, Mizutani E, Ohta H (2006). Significant improvement of mouse cloning technique by treatment with trichostatin A after somatic nuclear transfer. Biochem Biophys Res Commun.

[51] Bui HT, Wakayama S, Kishigami S (2010). Effect of trichostatin A on chromatin remodeling histone modifications, DNA replication, and transcriptional activity in cloned mouse embryos. Biol Reprod.

[52] Pasque V, Gillich A, Garrett N (2011). Histone variant macroH2A confers resistance to nuclear reprogramming. EMBO J.

[53] Pasque V, Halley-Stott RP, Gillich A (2011). Epigenetic stability of repressed states involving the histone variant macroH2A revealed by nuclear transfer to *Xenopus oocytes*. Nucleus.

[54] Mikkelsen TS, Hanna J, Zhang X (2008). Dissecting direct reprogramming through integrative genomic analysis. Nature.

[55] Kim K, Doi A, Wen B (2010). Epigenetic memory in induced pluripotent stem cells. Nature.

[56] Lister R, Pelizzola M, Kida YS (2011). Hotspots of aberrant epigenomic reprogramming in human induced pluripotent stem cells. Nature.

[57] Polo JM, Liu S, Figueroa ME (2010). Cell type of origin influences the molecular and functional properties of mouse induced pluripotent stem cells. Nat Biotechnol.

[58] Feng B, Ng J-H, Heng J-CD (2009). Molecules that promote or enhance reprogramming of somatic cells to induced pluripotent stem cells. Stem Cell.

[59] Lee J-H, Bugarija B, Millan EJ (2009). Systematic identification of cis-silenced genes by trans complementation. Hum Mol Genet.

[60] Lee J-H, Gaetz J, Bugarija B (2009). Chromatin analysis of occluded genes. Hum Mol Genet.

[61] Berthet C, Kaldis P (2007). Cell-specific responses to loss of cyclin-dependent kinases. Oncogene.

[62] Aladjem MI, Spike BT, Rodewald LW (1998). ES cells do not activate p53-dependent stress responses and undergo p53-independent apoptosis in response to DNA damage. Curr Biol.

[63] Mathieu J, Zhang Z, Zhou W (2011). HIF induces human embryonic stem cell markers in cancer cells. Cancer Res.

[64] Monk M, Holding C (2001). Human embryonic genes re-expressed in cancer cells. Oncogene.

[65] Wong DJ, Liu H, Ridky TW (2008). Module map of stem cell genes guides creation of epithelial cancer stem cells. Cell Stem Cell.

[66] Kim J, Woo AJ, Chu J (2010). A Myc network accounts for similarities between embryonic stem and cancer cell transcription programs. Cell.

[67] Palmer NP, Schmid PR, Berger B (2012). A gene expression profile of stem cell pluripotentiality and differentiation is conserved across diverse solid and hematopoietic cancers. Genome Biol.

[68] Sperger JM, Chen X, Draper JS (2003). Gene expression patterns in human embryonic stem cells and human pluripotent germ cell tumors. Proc Natl Acad Sci U S A.

[69] Marquardt JU, Raggi C, Andersen JB (2011). Human hepatic cancer stem cells are characterized by common stemness traits and diverse oncogenic pathways. Hepatology.

[70] Kim J, Orkin SH (2011). Embryonic stem cell-specific signatures in cancer: insights into genomic regulatory networks and implications for medicine. Genome Med.

[71] Holmberg J, He X, Peredo I (2011). Activation of neural and pluripotent stem cell signatures correlates with increased malignancy in human glioma. PLoS One.

[72] Ben-Porath I, Thomson MW, Carey VJ (2008). An embryonic stem cell-like gene expression signature in poorly differentiated aggressive human tumors. Nat Genet.

[73] Meyer N, Penn LZ (2008). Reflecting on 25 years with MYC. Nat Rev Cancer.

[74] Singh AM, Dalton S (2009). The cell cycle and Myc intersect with mechanisms that regulate pluripotency and reprogramming. Stem Cell.

[75] Smith KN, Singh AM, Dalton S (2010). Myc represses primitive endoderm differentiation in pluripotent stem cells. Stem Cell.

[76] Varlakhanova NV, Cotterman RF, deVries WN (2010). myc maintains embryonic stem cell pluripotency and self-renewal. Differentiation.

[77] Riggs JW, Barrilleaux BL, Varlakhanova N (2013). Induced pluripotency and oncogenic transformation are related processes. Stem Cells Dev.

[78] Egli D, Birkhoff G, Eggan K (2008). Mediators of reprogramming: transcription factors and transitions through mitosis. Nat Rev Mol Cell Biol.

[79] Halley-Stott RP, Pasque V, Astrand C (2010). Mammalian nuclear transplantation to germinal vesicle stage *Xenopus oocytes*—a method for quantitative transcriptional reprogramming. Methods.

[80] Byrne JA, Simonsson S, Western PS (2003). Nuclei of adult mammalian somatic cells are directly reprogrammed to oct-4 stem cell gene expression by amphibian oocytes. Curr Biol.

[81] Bhutani N, Brady JJ, Damian M (2010). Reprogramming towards pluripotency requires AID-dependent DNA demethylation. Nature.

[82] Chiu CP, Blau HM (1984). Reprogramming cell differentiation in the absence of DNA synthesis. Cell.

[83] Tsubouchi T, Soza-Ried J, Brown K (2013). DNA synthesis is required for reprogramming mediated by stem cell fusion. Cell.

[84] Koche RP, Smith ZD, Adli M (2011). Reprogramming factor expression initiates widespread targeted chromatin remodeling. Cell Stem Cell.

[85] Yamanaka S (2009). Elite and stochastic models for induced pluripotent stem cell generation. Nature.

